# Publication activities of German junior researchers in academic medicine: which factors impact impact factors?

**DOI:** 10.1186/s12909-016-0712-3

**Published:** 2016-07-25

**Authors:** Mona Pfeiffer, Martin R. Fischer, Daniel Bauer

**Affiliations:** 1 Institut für Didaktik und Ausbildungsforschung in der Medizin, Klinikum der Universität München, Ziemssenstr. 1, 80336 Munich, Germany; 2 Institut für Medizinische Lehre, Universität Bern, Konsumstr. 13, 3010 Bern, Switzerland

**Keywords:** Medical doctoral training, Publication productivity, Academic title Dr. med

## Abstract

**Background:**

Previous studies have shown medical students in Germany to have little interest in research while at the same time there is a lack of physician scientists. This study’s aim is to investigate factors influencing publication productivity of physicians during and after finishing their medical doctorate.

**Methods:**

We conducted a PubMed search for physicians having received their doctoral degree at Ludwig-Maxmilians-University Munich Faculty of Medicine between 2011 and 2013 (*N* = 924) and identified the appropriate impact factor (IF) for each journal the participants had published in. Gender, age, final grade of the doctorate, participation in a structured doctoral study program and joint publication activities between graduate and academic supervisor were defined as factors. For analyses we used nonparametric procedures.

**Results:**

Men show significantly more publications than women. Before their doctoral graduation men publish 1.98 (SD ± 3.64) articles on average, women 1.15 (±2.67) (*p* < 0.0001, *d* = 0.27). After completion of the doctorate (up to 06/2015), 40 % of men still publish, while only 24.3 % of women (*p* < 0.0001, φ = 0.17) continue to publish. No differences were found concerning the value of IFs. Similar results were found regarding the variable ‘participation in a structured doctoral study program’. Until doctoral graduation, program participants publish 2.82 (±5.41) articles, whereas participants doing their doctorate individually only publish 1.39 (±2.87) articles (*p* < 0.0001, *d* = 0.46). These differences persist in publication activities after graduation (45.5 vs. 29.7 %, *p* = 0.008, φ = 0.09). A structured doctorate seems to have positive influence on IFs (4.33 ± 2.91 vs. 3.37 ± 2.82, *p* = 0.006, *d* = 0.34). Further significant results concern the variables ‘final grade’ and ‘age’: An early doctoral graduation and an excellent or very good grade for the doctoral thesis positively influence publication productivity. Finally, joint publication activities between the graduate and his/her academic supervisor result in significantly higher IFs (3.64 ± 3.03 vs. 2.84 ± 2.25, *p* = 0.007, *d* = 0.28).

**Conclusions:**

The study’s results support the assumption about women’s underrepresentation in science as well as the relevance of structured doctoral study programs for preparing and recruiting young academics in medicine for scientific careers. Promoting women and further development of structured doctoral study programs are highly recommended.

## Background

In recent years, various studies have demonstrated and discussed the lack of interest in research and scientific careers among (prospective) physicians [1, 2] as well as a lack of physician scientists in Germany which will likely increase dramatically in the near future [3]. In Germany, the entry into scientific work is usually the medical doctorate. Since medical education in Germany does not require undergraduates to author final papers like master theses, the medical doctoral thesis is usually the very first independently written scientific paper in a doctor’s academic career. Unlike other academic disciplines, most doctoral candidates in medicine already begin work on their doctoral thesis during their medical studies. Partly as a result of this aspect, the relevance and quality of medical doctoral theses have been called into question in Germany for several years. In the last few years this discussion has grown to include demands for the integration of more research-oriented teaching in undergraduate medical training, the development of structured doctoral study programs and more possibilities to integrate research activities into the medical specialist training [4, 5]. Against this background, the purpose of this study is to investigate critical factors influencing publication productivity of physicians during their doctorate and in the first years afterwards. Scientific publications represent a visible result of scientific activities, which thus can be easily identified and measured. Therefore, publication productivity is a crucial and commonly used factor in measuring performance of scientists [6–11]. In the present study we operationalize publication productivity as an outcome variable through the number of publications as well as the related impact factors (IFs). We considered gender, age, participation in a structured doctoral study program, final grade of the doctorate and joint publication activities with the academic supervisor as potential moderating factors.

The study’s objectives are to determine,

1. Which factors influence the number of publications prior to (and including) the year of doctoral graduation?
2. Which factors influence publication activities from the first calendar year following doctoral graduation onwards?
3. Which factors influence the value of IFs of these publications?

On one side we conducted this study to learn more about the reasons for successful scientific publishing and on the other hand to derive specific recommendations about fostering junior researchers’ scientific productivity in academic medicine.

## Methods

### Sampling

The initial sample included 961 physicians who received their doctoral degree from Ludwig-Maximilians-University Munich (LMU) Faculty of Medicine between 2011 and 2013, based on data provided by the deanery. The LMU Faculty of Medicine is one of the largest of its kind in Germany with about 6000 students enrolled in various health study programs, with approx. 500 medical students graduating each year. Eight-hundred-eighty-one participants of the sample completed their medical studies at LMU, 80 participants came from other medical faculties for their doctoral thesis.

From the initial sample, we excluded 37 persons: 28 because the publications found in the PubMed-research could not clearly be attributed to them e.g. because of too common names, and nine because of pre-existing academic titles indicating an academic career before the doctorate and thus above-average amount of publications.^1^ The final sample included 924 physicians. Ethical approval for this study was granted by the faculty’s ethics board.

### PubMed search

We conducted a PubMed search for each participant, using the first name and surname and in a second, supplemental step searched for the surname combined with the first name initial. If results could be attributed to the participant (because of the subject area or matching co-authors), they were counted towards his or her publication list. For any inconclusive results, the search was extended by including the academic supervisor’s name (if available). When participants changed their name (e.g. due to marriage) before completion of their doctorate, we searched both names. Some publications were probably missed due to participants having changed their name after completion of their doctorate, most probably skewing data somewhat to the disadvantage of women. All kinds of publications (research articles, reviews, case reports, conference proceedings and letters) were included since all of them add to visibility in the scientific community. Since original articles are particularly important for scientific careers, e.g. for postdoctoral qualification in Germany (‘Habilitation’), we added separate calculations for original articles as well.

### Impact factors and subject areas

Despite many dissenting voices [12–14] using IFs of academic journals to measure and evaluate scientists and their scientific achievements is common practice. In the context of medical education research in Germany, Ziemann and Oestmann [15] used the number of publications and IFs to assess the quality development of medical doctoral theses at Charité Berlin over time. We made use of IFs in this study, though with certain modifications as described below. After the PubMed search we identified the appropriate IF for each journal the participants had published in, using the then current Thomson Reuters Journal Citation Report from 2013. An important criticism levelled at the use of IFs for evaluating scientific output relates to the problem that there are some subject areas with generally above average IF vs. subject areas with below average IF. The Association of the Scientific Medical Societies in Germany suggests using weighted IF based on the median of their respective subject areas [16, 17]. This subject-related IF is calculated by dividing an IF by the median of IFs within the respective subject area. In this study we calculated for all participants the means of their publications’ IF as well as means of the respective subject-related IF. For journals listed in several subject areas we choose the highest subject-related IF for further calculations.

### Outcome variables and factors

We defined the following outcome variables:

- The number of publications indexed on PubMed (total number of publications and lead authorships) released before and including the year of completion of the doctorate
- Publication activities from the calendar year following completion of the doctorate onwards (total number of publications and lead authorships). Due to the different doctoral graduation dates between 2011 and 2013 we just carried out a dichotomous analysis of *published* versus *not published*
- Total number of original articles and number of original articles as lead author
- Average IF and average subject-related IF.

The following attributes were set as factors:

- Final grade of the doctorate (excellent, very good, good and satisfactory)
- Gender
- Participation in a structured doctoral study program
- Age by time of doctoral graduation
- Joint publication activities between graduate and their respective academic supervisor (with regard to the outcome variables IF and subject-related IF).

It is assumed that there is a link between the final grade of the doctorate and the outcome variables since the doctorate regulations of LMU Faculty of Medicine require doctoral theses being published or at least deemed publishable to achieve the highest grades ‘excellent’ and ‘very good’ [18].

Various studies address gender issues in science. Women’s underrepresentation as authors of scientific publications was addressed among others by Rossiter [19] who first described the so called ‘Matilda effect of science’. This refers to ignoring scientific achievements of female researchers deliberately or involuntarily, according to the Matthew effect, which is commonly used in sociology of science and describes the phenomenon in which renowned researchers get more attention for their work than unknown researchers even if they carry out similar work [20]. Even today, women still publish less frequently as lead or last author (usually reserved for the senior researcher in medicine) than men and are moreover underrepresented as authors of single-authored papers [21]. Against this background, we will investigate gender differences in publication productivity among junior researchers at LMU Faculty of Medicine.

**Fig. 1:**
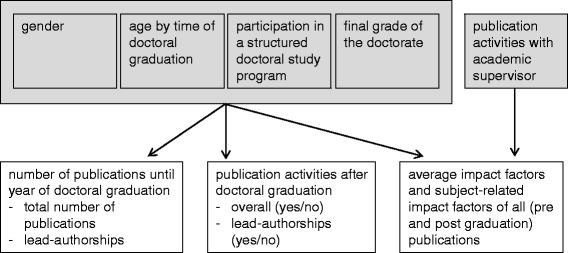
Assumed mode of action between variables

Not as frequently discussed as gender is the issue of structuring doctoral training. However, a few studies show positive effects of structured doctoral study programs on preparation for scientific careers [22] as well as for recruiting up-and-coming researchers in academic medicine [23]. Furthermore, an early doctoral graduation seems to have a positive impact on the future career of young scientists [24]. At least we assume a positive impact of joint publication activities between doctoral candidate/graduate and academic supervisor on IF since the importance of supervisors for a successful doctorate cannot be overstated [25]. Expected relationships between variables are represented in Fig. 1.

In the present study all variables were generated from data of the deanery or from search results of the PubMed-research. Thus they represent surrounding conditions or stable attributes like gender. Individual attributes like motivation or personality traits, which definitely having a strong impact on publication productivity, are not considered in this study.

### Data analysis

For analyses we used SPSS version 22 including descriptive analyses for mean, median, standard deviation (SD), maximal and minimum values as well as Mann–Whitney *U* Test, Kruskal-Wallis-H, chi-square-tests and Spearman’s Rho rank correlation. Since our data does not fulfil the qualifications for parametric procedures like regression analysis or ANOVA due to unequal sample sizes, no assumption of normal distribution and missing homogeneity of error variances, we decided on using nonparametric procedures.

The influence of dichotomous variables on continuous variables (total number of publications before and including the year of completion of the doctorate, number of lead authorships before and including the year of completion of the doctorate, total number of original articles and number of original articles as lead author, average IF and subject-related IF) was determined by using Mann–Whitney *U* test. As regards the variable ‘final grade of the doctorate’ which has 3 values, we used Kruskal-Wallis test calculating Mann–Whitney *U* test for post-hoc comparisons. In order to avoid multiple comparisons problems, the significance level was adjusted to 0.03 by Bonferroni-correction. Finally, we calculated Cohen’s *d* for effect sizes.

The influence of the variables gender, participation in a doctoral study program and final grade of the doctorate on the dichotomous variables publication activities after doctoral graduation (overall and lead authorships) was investigated by using chi-square tests. For effect sizes we calculated coefficient φ or Cramér’s V for final grade with 3 values.

Spearman’s Rho test was used to demonstrate correlations between participant’s age by time of doctoral graduation and continuous variables. In case of dependence between age and dichotomous variables (publication activities after graduation) Mann–Whitney *U* test was used.

## Results

### Sample

The mean age ± SD of the participants at the time of doctoral graduation was 31.97 (±5.26), minimum: 24.67 and maximum: 62.83 years. Five-hundred-thirty-nine (58.3 %) were female and 385 (41.7 %) male. The majority of the graduates did their doctorate individually (858, or 92.9 %) in contrast to only 7.1 % (66 participants) completing a structured doctoral study program (45.5 % female and 54.5 % male). Eighty-five participants (9.2 %) received the best grade ‘excellent’ , 485 (52.5 %) received the grade ‘very good’ , 327 received ‘good’ and 27 ‘satisfactory’. We combined these last two grades for further calculations, thus this group includes 354 participants (38.3 %).

Overall, 547 (59.2 %) of the participants authored 2462 publications between 01/2001 and 06/2015, whereas 377 graduates have never published (in journals indexed in Pubmed). These non-publishing graduates are on average 2 years older by the time of their doctoral graduation (33.58 vs. 31.60 years) than their publishing peers, have lower grades (2.67 vs. 2.08 on average), are more often female (64.5 %) and carried out their doctorate individually (97.1 %).

A few publications can be attributed to more than one participant in our sample as a result of students working in the same scientific working group. These 2462 publications spread over 55 different subject areas referring to Thomson Reuters Journal Citation Report 2013. The ten most frequent subject areas in which the participants of the present study published, the number of publications per subject area and the median IFs are represented in Table 1.

**Table 1:** Top ten subject areas with median IFs and number of publications per area (Thomson Reuters Journal Citation Report 2013)

Subject area	Median IF	Number of publications
Radiology, Nuclear Medicine & Medical Imaging	1.68	327
Surgery	1.37	254
Oncology	2.69	155
Cardiac & Cardiovascular Systems	2.20	129
Clinical Neurology	2.18	99
Gastroenterology & Hepatology	2.39	84
Orthopaedics	1.58	81
Urology & Nephrology	1.85	79
Medicine, Research & Experimental	2.15	77
Psychiatry	2.07	73

### Publication activities before and including the year of completion of the doctorate

The average number of publications until the completion of the doctorate ± SD was 1.5 (±3.14) per participant with a range between 0 and 43. The median was 0.5. The average number of publications as lead author was 0.32 (±0.94), range between 0 and 12, median: 0. Results for the variables gender, final grade of the doctorate and participation in a structured doctoral study program categorized by total number of publications and lead authorships are represented in Table 2 and described in the following paragraph.

**Table 2:** Total number of publications and number of publications as lead author until year of doctoral graduation

	Number of publications (total number)			Number of publications (lead authorships)		
Gender	male		female	male		female
Mean (SD)	1.98 (3.64)		1.15 (2.67)	0.42 (1.03)		0.25 (0.86)
Final grade of the doctorate	excellent	very good	good/satisfactory	excellent	very good	good/satisfactory
Mean (SD)	3.8 (5.68)	1.76 (3.13)	0.58 (1.57)	0.82 (1.38)	0.39 (1.05)	0.11 (0.46)
Participation in a structured doctoral study program	yes		no	yes		no
Mean (SD)	2.82 (5.41)		1.39 (2.87)	0.74 (1.51)		0.29 (0.87)

Men had a higher publication output, both in overall terms (1.98 ± 3.64) and in terms of lead authorships (0.42 ± 1.03) compared to women (total: 1.15 ± 2.67; lead authorships: 0.25 ± 0.86). This difference is statistically significant in the case of the total number of publications (*p* < 0.0001, *d* = 0.27) as well as in case of lead authorships (*p* = 0.001, *d* = 0.18).

The amount of publications dropped with decreasing final grade. Therefore, graduates with the best grade ‘excellent’ published 3.8 (±5.68) articles until graduation, whereas graduates with the two lowest grades ‘good’ and ‘satisfactory’ published only 0.58 (±1.57) articles. Lead authorships, showed very similar results. Post hoc-tests show significant differences between all groups (*p* < 0.0001). *d* assumes values between 0.33 and 1.13, with strongest effects between the groups ‘excellent’ and ‘good’/’satisfactory’.

Differences also appeared considering the variable ‘participation in a structured doctoral study program’. Hence graduates completing a doctoral study program on average published 2.82 (±5.41) articles, 0.74 (±1.51) of which are lead authorships. Graduates doing their doctorate individually only published 1.39 (±2.87) articles, of which only 0.29 (0.87) constitute lead authorships. In both cases, the differences are significant (*p* < 0.0001, *d* = 0.46 in case of overall publications, *d* = 0.48 in case of lead authorships).

Participants publishing before the completion of their doctorate were 30.95 (±4.05) years old on average by time of doctoral graduation and therefore 2.04 years younger than those publishing nothing up to the year of their doctoral graduation (32.99 ± 6.08). There is a small negative correlation (*r* = −0.13, *p* < 0.0001) between participants’ age and total number of publications until doctoral graduation. In view of lead authorships, the correlation is negligible (*r* = −0.08, *p* = 0.02).

### Publication activities after doctoral graduation

The majority of graduates (69.2 %) no longer published following completion of their doctorate. Differences in publication activities after graduation for the variables gender, final grade of the doctorate and participation in a doctoral study program are represented in Table 3.

**Table 3:** Publication activities after doctoral graduation

	Overall			Lead authorships		
Gender	male		female	male		female
Frequency	154		131	86		54
Percent within ‘gender’	40 %		24.3 %	34.3 %		18.2 %
Final grade of the doctorate	excellent	very good	good/satisfactory	excellent	very good	good/satisfactory
Frequency	45	190	50	26	89	25
Percent within ‘final grade’	52.9 %	39.2 %	14 %	32.9 %	25.5 %	21 %
Participation in a structured doctoral study program	yes		no	yes		no
Frequency	30		255	21		119
Percent within ‘participation in a structured doctoral study program’	45.5 %		29.7 %	36.8 %		24.3 %

Women published significantly less than men following doctoral graduation. Only 24.3 % of women were able to point to a minimum of at least one publication after doctoral graduation, while in contrast 40 % of men still published following doctoral graduation. This also applied in the case of lead authorships. Both results are highly significant (*p* < 0.0001, φ = 0.17 regarding overall publications and 0.18 in the case of lead authorships).

There are also similarities with publication activities until doctoral graduation considering the variables final grade of the doctorate and participation in a structured doctoral study program. Thus graduates with the grade ‘excellent’ published most frequently after their doctoral graduation (52.90 % overall and 32.90 % as lead author), however group differences only become significant regarding overall publication activities (*p* < 0.0001, Cramér’s V = 0.3).

Similarly, participation in a structured doctoral study program is also tied to higher publication rates. 45.5 % published as first- or co-author, contrasting with 29.7 % of graduates who did their doctorate individually (*p* = 0.008) and 36.8 % as lead author compared to 24.3 % of individual graduates (*p* = 0.04). Although both differences are significant, only low effect sizes are determined (φ = 0.09 for overall publications as well as lead authorships).

Graduates publishing after the completion of their doctorate were 30.56 (±3.91) years old on average at the time of their doctoral graduation and 2.04 years younger than those publishing no longer after completing their doctorate (32.60 ± 5.65). The age at doctoral graduation had a significant impact on overall publication activities (*p* < 0.0001, *d* = 0.39) and publication activities as lead author (*p* = 0.001, *d* = 0.36) after graduation.

### Publication types

Most of the 2462 publications are original articles (73.48 %). Other types include reviews, guidelines and meta-analyses (8.08 %), case reports (9.87 %), conference proceedings (5.08 %) and other types such as letters, comments, news, short communications, editorials and reports (3.49 %). Women publish significantly fewer (*p* < 0.0001, *d* = 0.42) original articles (2.47 ± 2.99) than men (4.30 ± 5.624). There are also significant gender differences concerning reviews (0.24 ± 0.7 vs. 0.51 ± 1.24, *p* = 0.001, *d* = 0,27) and case reports (0.29 ± 0.68 vs. 0.63 ± 1.39, *p* = 0.02, *d* = 0,32). No significant differences were found for the other publication types. Participation in a structured doctoral study program is tied to a higher number of original articles (5.52 ± 9.04 vs. 3.03 ± 3.47, *p* = 0.005, *d* = 0.56), however there is no significant difference regarding reviews, case reports, conference proceedings and other publication types.

Results concerning the total number of original articles and number of original articles as lead author are represented in Table 4 and described in the following paragraph.

**Table 4:** Total number of original articles and number of original articles as lead author

	Number of publications (total number)			Number of publications (lead authorships)		
Gender	male		female	male		female
Mean (SD)	4.30 (5.62)		2.47 (2.99)	0.84 (1.62)		0.45 (0.82)
Final grade of the doctorate	excellent	very good	good/satisfactory	excellent	very good	good/satisfactory
Mean (SD)	5.91 (8.15)	3.16 (3.45)	2.01 (2.80)	1.27 (2.25)	0.60 (1.04)	0.29 (0.69)
Participation in a structured doctoral study program	yes		no	yes		no
Mean (SD)	5.52 (9.04)		3.03 (3.47)	1.37 (2.57)		0.54 (0.96)

As previously described for overall original articles, men had also a higher publication output in terms of lead authorships (0.84 ± 1.62) compared to women (0.45 ± 0.82) which is statistically significant (*p* = 0.002, *d* = 0.31). Also considering the variable ‘participation in structured doctoral study program’ graduates doing their doctorate individually publish significantly fewer (*p* < 0.001, *d* = 0.67) original articles as lead author (0.54 ± 0.96) than those completing a program (1.37 ± 2.57).

The number of original articles (overall as well as lead authorships) increases with the final grade. Post-hoc tests reveal significant differences for all comparisons between groups (p ≤0.001) and *d* values between 0.32 and 0.7. There is a small negative correlation (*r* = −0.13, *p* = 0.004) between a participant’s age and the number of original articles as lead author whereas no correlation between age and the total number of original articles could be found.

### Impact factors and subject-related impact factors

The average IF was 3.47 (±2.84) for all graduates. The average subject-related IF was 1.74 (±1.34). There are no significant differences between men and women concerning the value of average IFs as well as subject-related IFs.

Participants with the best grade ‘excellent’ achieved the highest average IFs (4.49 ± 2.95) as well as subject-related IFs (2.15 ± 1.25). In both cases the three groups differ significantly (*p* < 0.0001) with effect sizes between *d* = 0.27 and 0.60 for single comparisons with highest effect sizes for comparisons between ‘excellent’ and ‘good’/’satisfactory’.

Furthermore, participation in a structured doctoral study program seems to have a positive influence on average IFs (4.33 ± 2.91 vs. 3.37 ± 2.82, *p* = 0.006, *d* = 0.34) as well as subject-related IFs (2.11 ± 1.28 vs. 1.7 ± 1.34, *p* = 0.007, *d* = 0.18).

Joint publication activities between the graduate and academic supervisor resulted in significantly higher average IFs (3.64 ± 3.03 vs. 2.84 ± 2.25, *p* = 0.007, *d* = 0.28) but not regarding subject-related IFs.

No significant correlations were detected between the graduates’ age and the value of average IFs as well as subject-related IFs. All results are represented in Table 5.

**Table 5:** Impact factors and subject-related impact factors

	Impact factors			Subject-related impact factors		
Gender	male		female	male		female
Mean (SD)	3.48 (2.67)		3.46 (2.99)	1.83 (1.28)		1.67 (1.39)
Final grade of the doctorate	excellent	very good	good/satisfactory	excellent	very good	good/satisfactory
Mean (SD)	4.49 (2.95)	3.51 (2.70)	2.70 (2.99)	2.15 (1.28)	1.77 (1.29)	1.41 (1.45)
Participation in a structured doctoral study program	yes		no	yes		no
Mean (SD)	4.33 (2.91)		3.37 (2.82)	2.11 (1.28)		1.70 (1.34)
Joint publications with academic supervisor (minimum 1)	yes		no	yes		no
Mean (SD)	3.64 (3.03)		2.84 (2.25)	1.80 (1.42)		1.56 (1.10)

## Discussion

### Gender differences in publication productivity

Women’s and men’s publication activities differ substantially. Even at a first glance at the data we noticed disproportional publication productivity between men and women: Our sample consisted of 41.7 % men and 58.3 % women but the gender ratio regarding publication activities is almost reversed. Overall publications until doctoral graduation consist of 46.1 % male and 53.9 % female authorships. This discrepancy increases further, with publication activities after doctoral graduation splitting 46 % female authorships vs. 54 % male authorships. Obviously, the smaller proportion of women publishing indicates that especially female physicians are not involved in scientific activities after their doctoral graduation, much less pursuing a scientific career. Furthermore, women publish less original scientific work, which is often crucial for postdoctoral career advancement, although we might have missed some publications due to name changes, allowing for some bias. This bias will probably persist in similar studies until such time that author identifiers (e.g. ORCID) have fully permeated the world of scientific publications. But our results still seem plausible enough, considering they are well in line with numerous studies concerning gender inequality in science. A recent study by Hill et al. [26] compares h-indices of women and men in academic gynaecologic oncology with the result that women, especially in their early career, have significantly lower publication outcomes than their male colleagues. This evens out during further career development [27, 28]. However women are significantly underrepresented in higher academic positions and there is evidence that women are less frequently recipients of research grants [29].

The reasons for women’s underrepresentation in science are discussed widely. Few studies referring to female researchers in medicine describe lower interest of women in science as possible reason as well as vocational barriers [30–32]. In this context, it is above all the lack of mentoring programs [30], discrimination against women [33, 34], as well as difficulties to combine family and career [35, 36] which are mentioned as reasons. There is some evidence that women’s academic career aspirations are fostered through positive contextual factors like a favourable academic environment or development meetings [37]. A current study by Knobloch-Westerwick et al. [38] demonstrates the Matilda effect experimentally, measuring attitudes towards male and female scientists’ scientific performance. Participants were given the task of reviewing conference abstracts which either were ostensibly written by men or women. Abstracts with assumed male authorship were attested higher scientific quality than those of assumed female authors. This result seems to be important above all concerning the increasing relevance of peer review but also in regard of assessment of academic achievements in general. So beyond mentoring and support programs, it is these (often implicit and potentially damaging) attitudes which need to be addressed. Men and women alike need to critically reflect in how far they might be prejudiced, belittling others or themselves. In this context, effective gender diversity trainings [39] are becoming available.

### The influence of structured doctoral study programs

Another important result of this study is the significantly higher publication outcome of participants of structured doctoral study programs. Graduates doing their doctorate individually publish less (2.41 ± 4.59) than graduates completing a doctoral study program (5.98 ± 11.82). This effect is apparent before as well as after doctoral graduation and for original articles and seems to be relevant for IF’s and subject-related IF’s value.

As elsewhere in Germany doctorate relationships at LMU Faculty of Medicine (but not only in medicine) are basically individual, which means the doctoral graduation is arranged according to a master-apprentice-model, and any student in the medical program can commence a doctoral thesis project.

To enter the structured doctoral study program, about 100 applicants compete for about 40 fellowships per year. With two exceptions the structured doctoral study program of the present study concerns the program “Förderprogramm für Forschung und Lehre – FöFoLe” offered by the LMU Faculty of Medicine concerning the subjects molecular medicine and systems biology [40]. This program is aimed at undergraduate medical students and offers monthly financial support, intensive supervision and accompanying courses and lectures.

Results of a study from 2011 demonstrate higher intrinsic motivation and interest in research of FöFoLe program participants compared to doctoral candidates doing their doctorate individually [41].

In German-speaking countries, doctoral study programs in academic medicine are considered crucial in counteracting the current and ever increasing lack of physician scientists [42]. As already mentioned, some recent studies show positive effects of doctoral study programs in medicine concerning young academics’ preparation for scientific careers [22] as well as for recruiting up-and-coming researchers in academic medicine [23]. However, it needs to be taken into account that the mentioned studies refer to MD-PhD programs whereas the FöFoLe program is solely for MD candidates. Nevertheless, the present findings support the assumption of positive consequences through participation in structured doctoral study programs. Although we know little about the actual professional careers of each program participant, higher publication productivity, especially after graduation as well as the significantly higher publication rate of original articles, which is important for postdoctoral careers, suggests that these graduates more often work in academic medicine and possibly pursue an academic career.

### Discussion of further results: age, doctoral thesis grades and support of academic supervisors

The doctorate’s final grades consistently significant influence on publication productivity appears obvious since the doctorate regulations of LMU Faculty of Medicine require doctoral theses being published or deemed publishable for the highest assessments ‘excellent’ and ‘very good’ [18]. However, the grading of one’s doctorate seems to be a good predictor for further publication activities after the doctoral phase.

Furthermore, an early doctoral graduation (and thus an early postdoc) has a small but positive effect on publication productivity. Similar effects were already described in a study by Horta [24]. In our case of academic medicine this fact possibly goes hand in hand with a high workload in the first years of the medical specialist training which may negatively affect scientific productivity.

The relevance of the support through academic supervisors is probably much more important than this study can demonstrate. For methodological reasons we can only demonstrate the existence of an impact of joint publication activities between graduates and academic supervisors on the impact factor values. But numerous other studies describe the significance of mentors for the development of scientific interest and pursuing a scientific career [1, 43].

### Limitations

The variables investigated in the present study are all based on information offered by the deanery of LMU Faculty of Medicine or were distilled from PubMed. Individual aspects such as scientific interests or motivation could therefore not be considered. Another problem concerns the difficulty in measuring scientific achievements. Parameters like number of publications and IFs as well as subject-related IFs are purely quantitative and do not say anything about the quality of the single publications. On the other hand, the discussion leads to numerous connecting factors concerning the improvement of medical doctorates’ training which can be initiated by medical schools. Without a doubt, the two most important tasks are the intensive promotion of women in medical science and development and expansion of structured doctoral study programs.

We would like to point out that the FöFoLe-Program funded this study. Although program representatitves have not influenced this study, potential sponsorship effects cannot be fully excluded.

Furthermore, the results are relevant with regard to the recent recommendations on the development of medical education in Germany of the German Council of Science and Humanities [44].

## Conclusions

More than half of the graduates in the present study published a minimum of one paper as an academic result of their doctoral thesis. Publishing such papers on the one hand makes the graduates’ research performance visible and prevents potentially interesting research results from gathering dust in inaccessible university archives where theses often end up. On the other hand, our data also suggests that many doctoral candidates are successfully integrated into the complete research process, which includes communication of research results to the scientific community as its final step – ideally in the form of peer-reviewed paper [45]. One third of the participants still publishes after completion of their doctorate, which suggests that they work in scientific contexts. However, the study’s results also demonstrate women’s underrepresentation concerning publication authorship in scientific medical journals. Although nowadays more than half of medical students in Germany are female, women publish significantly less than their male colleagues and hence seem not to pursue careers in academic medicine as often as male physicians. It is therefore highly recommended to specifically promote women in academic medicine for example through the development of special mentoring programs or by targeting women to participate in structured doctoral study programs. In our particular case, women in the FöFoLe program are obviously underrepresented. The main objective of promoting women in academic medicine is the goal of equal opportunities for men and women in science. But promoting female researchers in academic medicine is also necessary to address the lack of physician scientists in Germany. Development and expansion of structured doctoral study programs in medicine are equally important. The study’s results support the assumption regarding the programs’ role of preparing and recruiting young academics in medicine for scientific careers. The importance of further development of such programs and expansion to other medical specialties cannot be overestimated. Medical Faculties often benefit from the scientific work carried out by doctoral candidates, increasing their scientific reputation and to some extent performance-related resource allocation. As doctoral candidates are usually unpaid or not well paid, however, a desirable quid pro quo would be for medical faculties to provide the best possible supervision and working conditions for their junior researchers.

## Abbreviations

IF, impact factor; SD, standard deviation

## Footnotes

1 In seven out of these nine cases, participants hold the title of ‘Dr. med. univ.’ which is commonly used in Austria for practicing physicians. However, these so called professional doctorates do not count towards postdoctoral qualification (‘Habilitation’) in Germany, for which the German ‘Dr. med.’ is required.
